# New framework for rehabilitation – fusion of cognitive and physical rehabilitation: the hope for dancing

**DOI:** 10.3389/fpsyg.2014.01478

**Published:** 2015-01-28

**Authors:** Prabhjot Dhami, Sylvain Moreno, Joseph F. X. DeSouza

**Affiliations:** ^1^Department of Biology, York UniversityToronto, ON, Canada; ^2^Rotman Research Institute at Baycrest HospitalToronto, ON, Canada; ^3^Department of Psychology, University of TorontoToronto, ON, Canada; ^4^Department of Psychology, Centre for Vision Research, York UniversityToronto, ON, Canada

**Keywords:** neurorehabilitation, dance, combined therapy, plasticity, music therapy

## Abstract

Neurorehabilitation programs are commonly employed with the goal to help restore functionality in patients. However, many of these therapies report only having a small impact. In response to the need for more effective and innovative approaches, rehabilitative methods that take advantage of the neuroplastic properties of the brain have been used to aid with both physical and cognitive impairments. Following this path of reasoning, there has been a particular interest in the use of physical exercise as well as musical related activities. Although such therapies demonstrate potential, they also have limitations that may affect their use, calling for further exploration. Here, we propose dance as a potential parallel to physical and music therapies. Dance may be able to aid with both physical and cognitive impairments, particularly due to it combined nature of including both physical and cognitive stimulation. Not only does it incorporate physical and motor skill related activities, but it can also engage various cognitive functions such as perception, emotion, and memory, all while done in an enriched environment. Other more practical benefits, such as promoting adherence due to being enjoyable, are also discussed, along with the current literature on the application of dance as an intervention tool, as well as future directions required to evaluate the potential of dance as an alternative therapy in neurorehabilitation.

## INTRODUCTION

Neurological disorders have been estimated to affect as many as a billion people worldwide, with this number expected to increase in the upcoming years (World Health Organization, 2006). Such disorders can be heterogeneous in regards to their symptoms and can include any combination of impairments related to physical and cognitive functioning, or issues with behavior, all of which can impact the basic daily living capability of individuals. Unfortunately, there are no current treatments that can address all symptoms in a meaningful manner. Surgical and pharmacological therapies for prevalent neurological disorders, such as Alzheimer’s and Parkinson’s, have been developed, but may only address a subset of symptoms, and even then, do so with limited efficacy (Ahlskog, 2011; Ahlskog et al., 2011; Intlekofer and Cotman, 2013). Adjunct conventional rehabilitative programs have been used as part of treatment regimes, although the effectiveness of such conventional therapies may also be limited (Lincoln et al., 1999; Langhammer and Stanghelle, 2000; Woldag and Hummelsheim, 2002; Bassett, 2003).

Recently, scientists have highlighted that exercise and music related activities can induce neuroplasticity, and are capable of aiding with physical and cognitive impairments across various neurological patient groups, including in those suffering from dementia and Alzheimer’s (Heyn et al., 2004; Thompson et al., 2005; Irish et al., 2006; Bruer et al., 2007; Lautenschlager et al., 2008), stroke (Duncan et al., 1998, 2003; Gordon et al., 2004; Schneider et al., 2007; Särkämö et al., 2008; Quaney et al., 2009), and Parkinson’s disease (Thaut et al., 1996; Crizzle and Newhouse, 2006; Goodwin et al., 2008; Tanaka et al., 2009; Cruise et al., 2011). However, the uses of such therapies are also faced with inherent limitations, creating the need to explore for further options.

Here, we propose dance as an intriguing alternative to physical and musical therapies as used in neurorehabilitation. As a physical activity, dance may be able to aid with physical functioning. However, other elements found in dance may contribute to it being a cognitively stimulating activity as well. This may allow dance to have a positive impact on not only physical, but cognitive functioning as well, in part due to fitting the framework of what are known as combined, or multimodal, therapies, which incorporate simultaneous physical and cognitive activity in a stimulating environment (Lustig et al., 2009; Kraft, 2012). Dance may also be able to overcome some of the more practical limitations associated with other alternative therapies. In the following sections, the current literature on the use of physical and musical activities in neurorehabilitation is discussed, along with the limitations associated with such therapies, as well as how dance can be a potential alternative in neurorehabilitation.

## PHYSICAL EXERCISE AND NEUROREHABILITATION

Physical activity, particularly aerobic exercise, has recently drawn interest for its potential use in neurorehabilitation. Engagement in physical exercise has been commonly reported as being associated with a reduction in risk for various neurological disorders, notably for cognitive decline, dementia and Alzheimer’s (Larson et al., 2006; Hamer and Chida, 2009; Sofi et al., 2011; Buchman et al., 2012). There is also support linking engagement in physical exercise to a reduced risk for onset of Parkinson’s (Xu et al., 2010) as well as stroke incidence (Do Lee et al., 2003), although these findings are not as robust as those for dementia and cognitive decline. Such epidemiological related studies suggest exercise having a neuroprotective effect in relation to the onset of various neurological disorders (Hillman et al., 2008). However, these findings do not offer support for how physical exercise can be used an as intervention for those who already suffer from such disorders. Rather, the interest in using exercise for neurorehabilitation stems from training based animal and human studies, which have helped demonstrate the widespread impact exercise can have on cognition, plasticity and overall brain health, particularly on the aging brain.

Although not directly translatable to humans, studies in animals have helped elucidate the fine neural changes that occur in the brain as a result of exercise. The impact of exercise on neurogenesis has been one of the focal points of such studies. Although a decline in neurogenesis is associated with aging, one of the most consistent findings in animal models is an increase in neurogenesis in the dentate gyrus of the hippocampus as a result of exercise (Hillman et al., 2008; van Praag, 2008), which is associated with improvements in cognition, particularly in learning and memory (van Praag et al., 1999, 2005; Vaynman et al., 2004; van Praag, 2009). As for the clinical significance, it has been hypothesized that a reduction in neurogenesis, as found in Alzheimer’s (Hillman et al., 2008), may exacerbate memory impairments, possibly through interfering with hippocampal neural circuits (Lazarov et al., 2010); exercise may represent an endogenous approach for the restoration of cells in the hippocampal dentate gyrus. However, the impact of exercise on neurogenesis and how this relates to improving cognitive impairments remains unclear (van Praag, 2008, 2009; Lazarov et al., 2010; Mu and Gage, 2011).

Exercise can also impact brain vasculature, particularly by increasing angiogenesis throughout the brain (Cotman et al., 2007; Voss et al., 2013), including in regions such as the hippocampus (Kramer and Erickson, 2007), motor cortex (Kleim et al., 2002; Swain et al., 2003), and cerebellum (Black et al., 1990). Brain health can be impacted via new blood vessels being used to deliver necessary nutrients to both new and old neurons in the brain (Kramer and Erickson, 2007). Neurotrophic factors, which promote plasticity, such as insulin-like growth factor, fibroblast growth factor 2, and brain derived neurotrophic factors (BDNFs), have also been shown to be upregulated following exercise treatments (Cotman and Berchtold, 2002; Vaynman and Gomez-Pinilla, 2005). BDNF has been of particular interest due to its potential role in promoting neural reorganization and regeneration in an impaired central nervous system (Vaynman and Gomez-Pinilla, 2005). Its impact on neurorehabilitation may stem from its crucial role in hippocampal learning and memory formation through long term potentiation (Egan et al., 2003; Hillman et al., 2008), its involvement in neuroprotection and promotion of cell survival (Kramer and Erickson, 2007), as well as recovery of motor functions (Griesbach et al., 2004; Ploughman et al., 2009).

Intervention based human studies, with a focus on healthy older adults, have also been able to show that exercise training can enhance brain plasticity and cognition. In a meta-analysis, Colcombe and Kramer (2003) surveyed 18 studies that included randomized aerobic fitness training in older adults, and reported a moderate effect size for the impact of fitness training on cognition. Specifically, greater executive, controlled, spatial and speed processes were linked to fitness training, with executive control showing the largest effect size. Intervention based neuroimaging studies in healthy adults have also shown that physical exercise can lead to various changes in the brain, including increased functional activity in frontal and parietal regions and decreased functional activity in anterior cingulate cortex regions (Colcombe et al., 2004), increased gray matter volume in regions of the frontal and superior temporal lobe (Colcombe et al., 2006) as well as in the hippocampus (Erickson et al., 2011), and significant increases in the blood volume of the hippocampal dentate gyrus (Pereira et al., 2007). Such changes, particularly those in the hippocampus, have also been found to be correlated to improvements in cognition (Pereira et al., 2007; Erickson et al., 2011). Thus, both animal and human studies have helped elucidate the various ways in which exercise can have an impact on the brain.

In regards to application to neurological groups, exercise has been found to aid with physical and functional impairments, including in stroke (Duncan et al., 1998, 2003; Gordon et al., 2004), Parkinson’s (Crizzle and Newhouse, 2006; Goodwin et al., 2008), and dementia patients (Heyn et al., 2004). Some studies suggest that exercise can also improve cognitive functioning in neurological groups (Heyn et al., 2004; Quaney et al., 2009; Tanaka et al., 2009; Baker et al., 2010; Cruise et al., 2011; Nagamatsu et al., 2013), although the current evidence has also been viewed as being mixed (Angevaren et al., 2008; Forbes et al., 2008; Busse et al., 2009; McDonnell et al., 2011; Snowden et al., 2011). Reports of small effect sizes (Lautenschlager et al., 2008), gender effects (Baker et al., 2010), variance in what cognitive domains are reported to be improved (Angevaren et al., 2008), as well as methodological differences (McDonnell et al., 2011; Snowden et al., 2011), are current limitations as reported in the literature, making it difficult to interpret the potential efficacy of exercise in neurorehabilitation.

There may also be limitations associated with transferring the use of exercise to real world clinical applications. For example, some of the studies discussed used moderate to high intensity exercises (Lautenschlager et al., 2008; Baker et al., 2010; Cruise et al., 2011), although those with more severe symptoms may not be able to be active at such levels (Snowden et al., 2011). Also, adherence issues may impact the use of conventional exercise as a therapy. It has been estimated that over 50% of participants who begin an exercise program will drop out within the first 6 months (Dishman, 1988). This is particularly prominent in older adults, who may initially be willing to participate in an exercise program, but only do so for the short term, eventually stopping (Van Der Bij et al., 2002). Unfortunately, the benefits of exercise require continued participation, making the issue of adherence that much more important. Given that neurological disorders affect patients for the long term, it is important that any therapy used is capable of promoting continuous commitment that ends up becoming part of the person’s weekly routine. Various factors that may contribute to whether someone will continuously participate in an exercise program include social support, health, personal beliefs, as well as motivation and enjoyment (Rhodes et al., 1999). However, traditional exercise programs may not fulfill many of these needs; in particular, conventional methods of exercise may not be seen as enjoyable by participants, which can have a significant impact on adherence (Rhodes et al., 1999; Belardinelli et al., 2008; Findorff et al., 2009). Such issues are important to keep in mind when promoting the use of physical exercise for neurorehabilitation.

In summary, there is strong support from both animal and human studies that physical exercise can be beneficial for the brain. It is important to note that the vast majority of these studies are based on the use of exercise in healthy populations, where a major focus has been on its potential role in healthy aging. Unfortunately, the efficacy of exercise for aiding with cognition in neurological disorders remains difficult to interpret. Both animal and human clinical studies have yet to produce the robust findings of the impact of exercise as found in their healthy counterparts. Methodological differences found between studies also contribute to the effect of exercise being unclear. Other more practical factors, such as the intensity of exercise needed to produce meaningful results, as well as enjoyment and adherence issues, also pose potential limitations for the application of exercise in neurorehabilitation.

## MUSICAL ACTIVITIES AND NEUROREHABILITATION

Music has been described as a powerful multimodal stimulus in humans (Sacks, 2006), invoking the widespread activity of various brain regions related to sensorimotor, higher order cognitive and emotional processes (Menon and Levitin, 2005; Koelsch, 2009; Herholz and Zatorre, 2012). Such processes can include auditory processing, attention, memory and sensory-motor integration, leading to the involvement of networks that consist of frontal, temporal, parietal and subcortical regions (Zatorre, 2005; Schlaug, 2009). Music processing can also be quite a complex task, recruiting various brain regions that are associated with the different components found in music, including pitch, timbre, rhythm, melody, recognition, and emotion (Lin et al., 2011). Musical experiences have also been shown to extensively enhance brain plasticity across the lifespan, leading to alterations of structural and functional properties of the human brain (Gaser and Schlaug, 2003; Schlaug et al., 2005; Hyde et al., 2009; Herholz and Zatorre, 2012). The ability of music to stimulate the widespread activity of brain regions through the engagement of various processes, as well as being able to enhance neuroplasticity, has led to interest in how the effects of musical activities can be generalized beyond the musical domain, and be used to improve various neurological-related impairments (Schlaug, 2009; Thaut et al., 2009; Altenmüller and Schlaug, 2013; Hegde, 2014).

Engaging in musical activities has been found to have a powerful effect on cognitive functions across the lifespan. Although aging is associated with a decline in various cognitive and perceptual domains (Rowe and Kahn, 1997), engagement in music related activities at a younger age may be able to help mitigate some of these effects later on in life. For example, in older adults, prior musical experience has been associated with enhanced perception of speech in a noisy environment as well as auditory working memory capacity (Parbery-Clark et al., 2011), a delay in the age associated decline of neural encoding of speech perception in the brainstem (Parbery-Clark et al., 2012), as well as a greater preservation of cognitive functioning in domains such as non-verbal memory and executive processes (Hanna-Pladdy and MacKay, 2011). Engagement in musical activities throughout life may also be related to the prevention of gray matter decline in older age (Sluming et al., 2002), something that is associated with normal aging (Good et al., 2001). However, the benefits of music do not strictly apply to only those who have engaged in musical activities throughout their life. Instead, musical training has also been found to be a powerful intervention tool for improving cognition in healthy older adults (Bugos et al., 2007). Simply listening to music has also been shown to enhance cognition in both young (Thompson et al., 2001; Schellenberg et al., 2007) and older adults (Thompson et al., 2005), although such findings are attributable to the enhancement of arousal and mood, rather than music itself. With music being a powerful stimulus, music based activities have been viewed as holding potential for being an engaging and effective method of neurorehabilitation (Schlaug, 2009).

Musical therapies, in both active and passive forms, have been used in neurorehabilitation. For example, training in the playing of instruments has been used to improve motor movements, including range, speed and quality of movements, in stroke patients (Schneider et al., 2007). Such improvements were also found to be associated with increased activity of motor regions, as well as neural reorganization of the motor network (Altenmüller et al., 2009). The coupling of auditory-motor stimulation found in playing instruments may have a role in recovery of motor skills, with improvements possibly being associated with an increase in functional auditory-motor connectivity (Rodriguez-Fornells et al., 2012). The relationship between motor movement and auditory stimuli has also been the basis of rhythmic auditory stimulation, which consists of movements being done to rhythmic auditory cues, and has been found to lead to functional improvements in patients with brain injuries (Hurt et al., 1998) and Parkinson’s (Thaut et al., 1996), with it being particularly effective in aiding with gait and upper extremity functioning (Thaut and Abiru, 2010). Music therapies have also been developed for speech and language impairments, such as Melodic Intonation Therapy, which has been used to improve speech in non-fluent aphasics by having patients sing phrases in a manner that exaggerate the melodic content of normal speech, all while tapping in synchrony to the syllables with their left hand (Schlaug et al., 2010). Preliminary findings from general music therapies, which consist of participating in various musical related activities, have also shown to improve executive functioning in those with brain injuries (Thaut et al., 2009). Thus, various types of musical therapies have been shown to be of use in different clinical groups.

Listening to music can lead to the engagement of various sensorimotor, cognitive and emotional processes in the brain (Zatorre, 2005; Koelsch, 2009), leading to widespread activity of temporal, frontal, parietal, subcortical, and cerebellar regions (Särkämö and Soto, 2012). With music listening being an engaging and cognitively stimulating activity, it has also been explored as a potential tool for neurorehabilitation. Särkämö et al. (2008) investigated the effects of passive music exposure on cognition and mood in stroke patients. Patients were placed into a music listening group, a language group, or a control group, which consisted of listening to self-selected music, audio books or nothing, respectively. Music listening was found to enhance cognitive recovery, providing greater improvements in focused attention and verbal memory, in comparison to the language and control group. Improvements were also noted for depression and mood as a result of music listening. However, whether such effects were specific to music, were primarily influenced by arousal, or can have a long lasting impact remains unclear (Särkämö et al., 2008). A more recent study by Särkämö et al. (2014) showed that listening to music can lead to structural changes in the brain of stroke patients, particularly leading to an increase of gray matter in frontolimbic brain areas; these structural alterations were also found to be correlated with improvements in cognition. Music listening has also been associated with improvements in visual awareness and attention in patients with neglect (Soto et al., 2009; Tsai et al., 2013), aiding with memory in those with Alzheimer’s (Simmons-Stern et al., 2010), as well as improving cognitive performance in those with dementia related impairments (Thompson et al., 2005; Irish et al., 2006; Bruer et al., 2007).

Although there is no clear answer as to how music listening can provide therapeutic value, certain mechanisms have been proposed to be of potential importance. Music has been shown to be a powerful emotional stimulus, capable of modulating emotional and reward related systems (Blood and Zatorre, 2001; Brown et al., 2004; Menon and Levitin, 2005). This effect may be able to improve mood and arousal in patients, which may in turn positively impact how they react to therapy and rehabilitation (Van de Winckel et al., 2004). Listening to pleasurable music may also be able to enhance cognitive functioning through modulating the release of dopamine (Särkämö and Soto, 2012). This may be inferred based on the increase of dopamine being associated with various improvements in cognition (Husain and Mehta, 2011), as well as emotional arousal during music listening being found to be associated with dopamine release (Salimpoor et al., 2011). The potential long term effects of music listening may be related to how it may enhance neuroplasticity, which may include promoting neurogenesis in the hippocampus (Kim et al., 2006), increasing BDNF levels in the hypothalamus (Angelucci et al., 2007a) and hippocampus (Angelucci et al., 2007b), as well as providing an auditory enriched environment which can positively impact auditory cortical functioning (Engineer et al., 2004). It has also been speculated that listening to music can enhance the regeneration and repair of neurons through influencing the secretion of specific steroid hormones (Fukui and Toyoshima, 2008). Thus, although arguably the simplest form of a musical experience, just the act of listening to music may have a powerful influence.

Other properties of music which may also further enhance its therapeutic value include its potential influence on neurochemical changes across various domains, including stress and arousal (Chanda and Levitin, 2013), or how musical therapies can be seen as enjoyable activities, in part due to the presence of music, which can improve mood and motivation, and thus the efficacy of interventions (Schneider et al., 2007; Herholz and Zatorre, 2012). However, there are limitations to the application of music in neurorehabilitation. Passive music therapies when applied to groups with cognitive or behavioral issues may offer little support to improve general physical functioning, which although may not be the primary symptoms, can still be impacted. Active musical therapies may aid with physical symptoms, but they are often reported as being used to assist with very specific motor impairments, tailored to the needs of the patient, and lacking any impact on broader general functioning. The efficacy of musical activities, particularly that of passive listening, in aiding with cognitive impairments also remains unclear, with methodological issues in current studies cited as making it difficult for any clear-cut conclusions to be drawn (Vink et al., 2003). There also may be more practical issues, such as hearing impairments amongst the elderly (Gordon-Salant, 2005), which may impact therapies that require music listening, or the use of instruments, and expecting patients to learn to play in active musical training therapies. There are also questions about the long-term impact of music listening on cognition, and the specific cognitive domains music listening may be able to aid with (Särkämö et al., 2008). That is not say that listening to music cannot improve cognition, but rather there is a gap between the understanding of music’s potential role in improving cognitive impairments, and the existence of meaningful results in the widespread application of music to various neurological populations (Hegde, 2014). Thus, although musical therapies have provided promising results, their use as a neurorehabilitative tool for aiding with various neurological-related symptoms, particularly that of cognitive impairments, requires further investigation.

## DANCE AND NEUROREHABILITATION

### WHAT IS DANCE

Dance may be defined as the act of one or more bodies moving in a rhythmic manner cued by music. However, even in its simplest form, dance requires a complex and simultaneous engagement in both physical and cognitive domains. The specific elements of dancing can vary greatly, but common features include learning new sequences of movements and rehearsing them, music accompaniment, and typically being held in a group, fostering social interaction. Comparisons with expert dancers have also helped demonstrate training effects of dancing, which includes effects on specific memory domains and tasks (Starkes et al., 1987; Smyth and Pendleton, 1994; Hüfner et al., 2011), as well as with physical fitness properties such as posture control (Simmons, 2005; Rein et al., 2011) and balance (Crotts et al., 1996; Gerbino et al., 2007; Bruyneel et al., 2010). Expert dancers have also been found to have structural differences in sensorimotor networks (Hänggi et al., 2010) and the hippocampus (Hüfner et al., 2011), as well as functional neural differences related to their training, such as when visualizing movement to familiar versus unfamiliar music (Olshansky et al., 2014), supporting the notion that training in dance can induce specific neuroplastic changes. This ability for dance to be both a physical and cognitive engaging activity may hold value for its potential as an alternative therapy for various clinical groups. The use of dance for aiding with physical functioning will be discussed, but here we focus on a lesser explored topic, that of which how dance may also be able to aid with cognition.

### INTEREST IN DANCE FOR NEUROREHABILITATION

#### Combined training

Physical and cognitive training on their own have been shown to be useful to some extent for improving cognition, but there may be added benefits to combining the two into a single activity. It has been proposed that exercise may need to be done in a cognitively stimulating context in order to maximize its impact on neuroplasticity and cognition (Fabel and Kempermann, 2008). In regards to how such combined interventions can lead to greater functional benefits than physical or cognitive activity alone, animal studies suggest a synergistic effect, possibly due to differences in how physical and cognitive activity induce neuroplasticity. For example, both physical and cognitive activities have shown to increase neurogenesis in the hippocampus (Kempermann et al., 1997; van Praag et al., 1999), as well as improve learning and memory for hippocampal related tasks (Olson et al., 2006). However, the way in which they increase neurogenesis may be quite distinct, with exercise leading to an increase in neurogenesis by increasing precursor cell proliferation, where as an enriched environment promotes the survival of new cells (Kempermann et al., 2010). Fabel et al. (2009) found that in mice, physical exercise followed by cognitive stimulation through an enriched environment resulted in an additive effect. It is thought that although physical exercise can increase the precursor cell pool, cognitive stimulation may increase the recruitment of cells to be integrated into functional networks (Fabel and Kempermann, 2008). Animal studies also show support for combined physical and cognitive activities being able to significantly enhance learning and working memory abilities compared to either activity done alone, independent of exercise intensity or duration (Langdon and Corbett, 2012).

Although the literature for the explicit use of combined training in humans is limited, results from these studies are nonetheless promising. Combined training programs have been reported as providing significantly greater improvements in older adults across various cognitive functions compared to isolated physical and cognitive training programs (Fabre et al., 2002; Oswald et al., 2006). A more recent study by Anderson-Hanley et al. (2012) aimed at comparing the effects of physical exercise in a standard environment versus that in a cognitively stimulating environment. The study consisted of participants being placed into either a standard group, which consisted of stationary cycling in a traditional fashion, or in an experimental group, which consisted of stationary cycling with a virtual reality display. Although participants did not engage in any explicit cognitive tasks, the use of virtual reality in the context of exercise may be seen as cognitively stimulating since it would putatively recruit additional neural networks. Executive functioning was found to be significantly better in the virtual reality group compared to the standard group. Those in the virtual reality group were also found to have a significantly greater increase in BDNF levels. However, exercise related effort and fitness between the two groups were similar, suggesting that not physical exercise, but rather the combined physical and cognitive activity, led to the greater improvements in cognition found in the experimental group. Combined therapies have also been found to be effective in improving cognition in neurological groups (Suzuki et al., 2012; Coelho et al., 2013), although it is important to note that such studies only compared the combined group to a control group, making it impossible to evaluate the impact of the combined therapies in comparison to physical or cognitive only activities. Thus, although further investigation is required, preliminary findings from both human and animal studies suggest that there may indeed be additional benefits to combined therapies.

#### Dance as a combined intervention

Dancing may be considered an ideal example of what multimodal training should consist of, due to combining physical and cognitive activity together (Fissler et al., 2012; Kraft, 2012; Olsson, 2012) in an enriched environment (Kattenstroth et al., 2010). Aside from the physical activity that dancing requires, it also involves various cognitive functions such as perception, emotion, executive functioning, memory and motor skills (Foster, 2013). Indeed, neuroimaging studies have shown widespread activity in the brain during rehearsal of dance movements (Brown et al., 2006). The engagement of such a wide variety of cognitive faculties may be attributed to dance being an experience that provides multisensory stimulation in an engaging environment, due in part to incorporating components such as physical activity, music listening and social interaction together (Johansson, 2012). How dance engages various cognitive faculties may be best answered by examining the effects of dance training on experts.

As previously described, training in dance requires substantial physical and cognitive engagement. Dancing consist of being aware of one’s appropriate sequence of movements over time, as well as how these movements should be conducted in relation to external cues (Sevdalis and Keller, 2011). This may be evident by the potential training effects found in experts in comparison to non-dancers on specific cognitive tasks, including greater recall for structured sequences (Starkes et al., 1987), longer memory spans compared for ballet and nonsense movements (Smyth and Pendleton, 1994), as well as significantly greater performance in non-spatial memory tasks (Hüfner et al., 2011). Learning sequences of movements may also be enhanced in a dance setting through observation of dynamic kinematic information provided from other dancers during learning (Gray et al., 1991). Brain activity in response to action observation has also been implicated in dancing, with experts being shown to have an increase in activity in brain regions considered a part of the human mirror system when viewing well known movements in relation to their learning of dance (Calvo-Merino et al., 2005; Cross et al., 2006). Also, the neural substrate of learning to dance through effective observation may be similar to that of learning by physical practice (Cross et al., 2009), with speculation that the effective observational learning associated with dance may influence other cognitive skills as well (Kattenstroth et al., 2013). Other factors that may also enhance the ability of dance to be a cognitively stimulating activity may include its social nature. The social environment dancing provides may be a source of cognitive stimulation, where simple exercises may recruit a more widespread brain network if done with other people in comparison to alone (Saarela and Hari, 2008). However, two other sources of substantial cognitive stimulation in dance include the musical element it incorporates, as well as the complex physical and motor skills it calls upon, both of which will be expanded upon.

***Music in dance.*** Dance is undoubtedly a complex task, made of numerous smaller parts that come together in sequence. However, a particular feature of dance that may bolster its therapeutic value is its incorporation of music. As already discussed, music is a powerful source for auditory stimulation with an ability to engage numerous emotional and cognitive faculties (Menon and Levitin, 2005; Peretz and Zatorre, 2005), and it may be speculated that the simple exposure to music may play a big role in dance being a cognitively stimulating activity. However, in combined interventions, cognitive and physical stimulation are seen as having an interaction effect, where the benefits gained from combining the two outweigh the benefits from either activity done alone. Thus, in the context of dance as a combined therapy, it is important to address whether music can enhance the benefits already found in physical activity.

Exercise conducted with music has been shown to lead to significant improvements in cognition in various clinical groups (Emery et al., 2003; Van de Winckel et al., 2004), but whether physical activity with music can lead to greater improvements than exercise alone is still not clear. However, a recent study by Satoh et al. (2014) seems to suggest that there may be indeed an additive affect on cognition when the two are combined. Satoh et al. (2014) placed older healthy adults into either a control group, an exercise group, or an exercise with musical accompaniment group. The physical routine between the two exercise groups was identical, with the only difference being the musical element found in the combined group. Cognitive improvements were greatest in the combined music and exercise group, with visuospatial functioning found to be significantly greater in the combined group than in the exercise alone group. It was suggested that the findings may be explained in the multimodal framework, where the addition of music to exercise could have led to simultaneous physical and cognitive stimulation, producing greater cognitive improvements than exercise alone. Such findings may give credence to music alone being able to provide enough cognitive stimulation where it is able to enhance the effects of exercise. These findings may also be seen as providing additional support that dance is a multimodal stimulation. Aside from passively listening to music, the act of timing and synchronizing movements to music may also be cognitively demanding (Bläsing et al., 2012). In comparison to the lack of music, dancing with music has been shown to activate unique regions including the anterior vermis of the cerebellum, putamen and the medial geniculate cortex, regions believed to be involved in synchronizing movement to musical rhythms during dance (Brown and Parsons, 2008). Dancing may also be viewed as a dual tasking activity, with the need for attention to be divided amongst various components, such as navigation and balance (Hackney et al., 2007); music may also play a factor in this. In contrast to listening to a piece of music, which can consist of a state of undivided attention, dance requires not only the listening and processing of music, but also moving accordingly to the music being listened to, which may present another form of dual tasking. Indeed, musicians have been shown to outperform non-musicians on dual task performance, potentially in part due to the fine motor coordination musicians require while simultaneously processing various musical elements (Moradzadeh et al., 2014). Dance, although perhaps to a lesser extent, can also be seen as requiring fine physical and motor coordination being done to music, and thus may contribute to dance providing an exercise in dual tasking. As previously described, the impact of physical activity on cognition may be greatest when done with complex cognitive stimulation (Fabel and Kempermann, 2008). Music may act as such a source of stimulation, with its ability to engage various cognitive faculties and induce widespread activity throughout the brain (Zatorre, 2005; Schlaug, 2009), as well present a form of dual tasking. Thus, when portraying dance as a combined therapy, its incorporation of music may be just one, albeit very powerful, source of cognitive stimulation.

***Physical and motor skills in dance.*** In a multimodal framework, physical activity is used to place the brain in a plastic state, preparing the brain to respond to cognitive stimulation, and thus allowing for a greater impact of cognitive activities (Hotting and Röder, 2013). However, the physical activity itself may also be a source of cognitive stimulation. The question then becomes what physical activities should be used in combined therapies. Aerobic exercises, such as running, are a popular choice, but due to some of the more complex movements, as well as the learning that is required for such movements, dance may offer a unique method of physical exercise to not only induce neuroplasticity, but also enhance cognition.

More conventional aerobic exercises, such as walking, have been used with success in multimodal training (Fabre et al., 2002; Anderson-Hanley et al., 2012; Suzuki et al., 2012; Coelho et al., 2013). Changes to cardiorespiratory fitness, as a function of aerobic exercise intensity, are believed to contribute to aerobic exercise being able to provide improvements in cognition (Hayes et al., 2013). However, dance may not hold the same intensity as other aerobic exercises, particularly for older adults. For example, Kattenstroth et al. (2013) reported no improvement in cardiorespiratory performance in older healthy adults after attending weekly dance classes for 6 months. This was attributed to the low intensity nature of the dance class, which consisted of many disruptions during dancing to provide new instructions, thus preventing any continuous activity to take place for an extended period of time. Yet, even if low intensity dancing is not able to improve cardiorespiratory performance in older adults, it may still be able to positively impact cognition. It has been noted that results from the literature do not support the notion that exercise can lead to improvements in cognition only if cardiovascular fitness is significantly impacted (Etnier et al., 2006; Busse et al., 2009). Rather, physical activity, even at a low intensity where there is no significant change to cardiorespiratory fitness, may still be able to positively impact cognition and brain structure (Ruscheweyh et al., 2011; Hayes et al., 2013). Low intensity exercise has also been noted to still be able to enhance neuroplasticity, for example, increasing BDNF levels (Soya et al., 2007; Aguiar et al., 2011; Vaughan et al., 2014). The ability for low intensity activity to still have a positive impact on the brain may help explain why Kattenstroth et al. (2013) found no changes to cardiorespiratory fitness, but still reported significant improvements in various cognitive and sensorimotor domains as a result of dancing. That is not to say that dancing is incapable of leading to significant changes in cardiorespiratory fitness, since it has been shown to do so (Hopkins et al., 1990). Rather, low intensity dancing, regardless of whether it provides significant changes in cardiorespiratory fitness, may still be able to positively influence brain plasticity and cognition in a similar manner to other aerobic exercises. Yet, there may be other unique properties of dancing not found in other conventional aerobic exercises that may lead it to providing a potentially greater impact on enhancing cognition.

Dance can be viewed as a multifaceted activity, including not only physical fitness, but also motor fitness, which accounts for components such as balance, flexibility, speed and coordination (Voelcker-Rehage et al., 2010). The extensive engagement in motor fitness may be evident by trained dancers having been shown to be significantly better than non-dancers across a variety of motor fitness domains, including posture control (Simmons, 2005; Rein et al., 2011) and balance (Crotts et al., 1996; Gerbino et al., 2007; Bruyneel et al., 2010). Expert dancers have also been shown to have a structurally altered sensorimotor network, including regions such as the premotor cortex, supplementary motor area and putamen (Hänggi et al., 2010). These alterations have been speculated to be related to dancing requiring intensive training in motor skills such as precise posture control, as well as coordination of the body for both gross and fine motor movements (Hänggi et al., 2010). Dancing has also been found to elicit significant improvements in various parts of motor fitness in older adults (Hopkins et al., 1990; Shigematsu et al., 2002; Verghese, 2006; Keogh et al., 2009; Kattenstroth et al., 2013). There is also evidence to suggest that both physical and motor fitness in older adults are positively associated with cognition, but the two differ with respect to the specific cognitive domains that they are associated with (Voelcker-Rehage et al., 2010). Exercises that combine physical and motor fitness have also been found to lead to significant improvements across a variety of cognitive domains in older adults (Vaughan et al., 2014). Coordination related exercises have revealed to require greater attention and concentration during execution compared to simple physical tasks (Mochizuki and Kirino, 2008). These findings do not necessarily suggest that one of motor and physical fitness exercises is in a sense better than the other. Rather, each may provide its own unique benefits, leading to the speculation that exercises that combine both may have a greater impact on cognition than those that only involve either type of fitness. In this context, dance may be an ideal activity due to requiring participants to extensively engage in both physical and motor fitness, and thus may be more encompassing in regards to employing different cognitive domains in comparison to other conventional exercises.

The difficulty of a physical activity and its requirement for novices to learn new physical skills may also be a factor in the potential neuroplastic changes induced by dance. Black et al. (1990) found that in rats, acrobatic training led to greater synaptogenesis in the cerebellar cortex than simple physical exercise training. This was attributed to the acrobatic training consisting of learning new and difficult motor tasks, whereas the physical exercise consisted of simple and repetitive activity of an already well known task (walking). Similar findings were reported by Curlik et al. (2011), who investigated the effects of physical activity that require skill learning on the survival of cells in the adult hippocampus. Rats were placed into separate groups, consisting of no learning (sedentary), physical skill learning and exercising. The exercise group consisted of standard wheel running. In comparison, the physical skill-learning group involved rats having to learn to stay on a rotating rod, with the speed of rotation gradually increasing over time, thus increasing the difficulty of the task as well as creating a need to adjust and relearn. Rats that successfully learned to stay on the rod were found to retain more neurons in their hippocampus than rats from the other groups, even though the exercise group ran twenty times the distance of the learning group. Furthermore, the survival rate of neurons was found to be similar in the exercising and no learning group. The findings indicated that cell survival was related not to the amount of exercise, but to complex skill learning. Based on these findings, Curlik and Shors (2013) suggested that exercises that combine mental and challenging physical skill learning, such as dance, could lead to long lasting effects on the adult brain. Findings with older human adults also show support for the complexity of the physical components of an exercise affecting cognitive outcomes. In humans, coordination exercises have been shown to have a greater positive impact on improving attention and concentration than simple aerobic exercises, possibly due to the complexity of the coordination exercise engaging neural networks that are not recruited in aerobic exercises (Budde et al., 2008). Such findings may also extend to dance when being compared to other exercises. For example, Coubard et al. (2011) reported that dancing had a significant effect on attentional control in older healthy adults, whereas Tai Chi and fall prevention exercises failed to have any effect. These findings were attributed to the dance intervention consisting of improvised movement, requiring participants to consistently adapt to new motor movements, and thus requiring higher attentional demand, all in contrast to the other two exercises. The inclusion of novel and difficult movements in dance therapy has been suggested to be helpful with improving certain physical impairments found in neurological disorders such as Parkinson’s (Duncan and Earhart, 2012). Physical exercise is undoubtedly important, but doing so in a simple and non-stimulating environment may limit its potential in improving cognition (Horne, 2013). Rather, activities, such as dancing, may be able to provide physical novel learning experiences in a stimulating context.

Aside from being a physical activity that includes extensive motor training and novel physical skill learning, dance may also incorporate other elements that can positively influence the impact of a physical activity on neuroplasticity and cognition, such as fostering social interactions. Simply walking with other people has been shown to activate a more widespread brain network compared to walking alone (Saarela and Hari, 2008). Social isolation during running has also been shown to suppress neurogenesis (Stranahan et al., 2006). Yet, social interaction is rarely lacking in dance, due to the two being intrinsically tied to one another. Dancing is most often done with others, either in pairs or groups, with individuals often relying on others for movement cues. However, going even farther back, dance has been speculated to have originated due to the need for social interaction, acting as a means of non-verbal communication (Brown and Parsons, 2008; Kattenstroth et al., 2013). Ultimately, dance incorporates various factors which may enhance a physical exercise’s influence on neuroplasticity and cognition, such as motor training, novel physical skill learning, and social interactions.

#### Other potential benefits of dance

Thus far, the potential use of dance for neurorehabilitation has focused on how it simultaneously combines physical and cognitively stimulating activities into one. However, there are other elements of dance that may also contribute to its rehabilitative properties. For example, in regards to the setting of rehabilitation, interventions may be most effective when done in an engaging environment that provides novel and multisensory stimulation (Maegele et al., 2005; Fabel and Kempermann, 2008; Kempermann et al., 2010; Pekna et al., 2012). Animal models support the ability of enriched environments to enhance neuroplasticity through various mechanisms (van Praag et al., 2000), improve cognitive deficits across a variety of neurological disorders (Pang and Hannan, 2013), as well potentially be a more potent therapy for cognitive impairments than physical exercise alone (Will et al., 2004). The very nature of dancing incorporates physical, cognitive and social engagement into a single setting, rich in components that touch upon all these domains; such components include rhythmic motor coordination, balance, memory, emotional engagement, affection, social interaction, acoustic stimulation as well as a musical experience, all of which add up to making dance a human equivalent of an enriched environment (Kattenstroth et al., 2010).

The value of dance as a tool for neurorehabilitation may also extend beyond its potential to improve physical and cognitive impairments. For example, depression is a common comorbid condition associated with various neurological disorders (Rickards, 2005; Hellmann-Regen et al., 2013), capable of not only exacerbating existing symptoms, but also impeding the effects of therapies being undertaken, ultimately reducing quality of life. However, participation in dancing has been shown to be able to significantly improve depression related symptoms (Kiepe et al., 2012; Koch et al., 2014; Vankova et al., 2014). Dancing may also be able, to a certain extent, avoid the adherence issues associated with conventional physical rehabilitation exercises (Dishman, 1988; Van Der Bij et al., 2002; Bassett, 2003). For example, whether an exercise is enjoyable and interesting can influence long term participation (Rhodes et al., 1999; Findorff et al., 2009), something that conventional exercises may not promote. In contrast, dance has been reported as being a highly enjoyable and motivating social activity, leading to adherence in both healthy and clinical groups (Federici et al., 2005; Belardinelli et al., 2008; Westheimer, 2008; Earhart, 2009; Hackney and Earhart, 2009; Heiberger et al., 2011; Houston and McGill, 2013). Factors found in dance that may contribute to this may include it being a social activity; group based exercises have been reported to possess higher participation rates (Van Der Bij et al., 2002), which may in part be explained by the act of developing social relationships being associated with the enjoyment of an exercise (Wankel, 1985). In addition, activities that are seen as requiring low exertion are favored by older adults (Rhodes et al., 1999). Music also has been noted to enhance participation in exercise, due to lessening the perceived difficulty and discomfort associated with physical activity (Johnson et al., 2001; Schutzer and Graves, 2004).

Thus, dance may be viewed as an interesting alternative therapy for neurorehabilitation due to how it combines various elements into a single experience, including cognitive and physical activities, emotional engagement, social interactions and multisensory stimulation (Kattenstroth et al., 2010), each of which may contribute to its therapeutic value.

### CURRENT EVIDENCE FOR THE USE OF DANCE

In regards to investigating the benefits of dance, its use in improving physical functioning has been the focal point. The various physically related benefits that dancing can provide, particularly for older adults, are well known, and includes improving balance, postural control, endurance and motor performance (Hopkins et al., 1990; Shigematsu et al., 2002; Federici et al., 2005; Verghese, 2006; Hui et al., 2009; Keogh et al., 2009; Kattenstroth et al., 2013). These improvements may be attributed to dance being an incredibly comprehensive exercise, including components of both motor and physical fitness, which may allow it to improve flexibility, heighten proprioception, improve muscle strength, and promote balance and posture (Westheimer, 2008). However, a limited number of studies involving both healthy and clinical groups suggest that dancing may also be able to improve cognitive functioning.

In an observational study, Kattenstroth et al. (2010) compared older healthy adults who had on average over a decade’s worth of experience in amateur dancing to control matched adults. The amateur dancing group was found to have a superior performance across numerous sensorimotor and cognitive domains in comparison to the no dancing group. However, an important observation was that in relation to the numerous domains that were tested, the difference between the two groups was related to the control group showing poor performances across multiple parameters, and not necessarily the dancing group exhibiting superior performances. This was interpreted as engagement in dance preventing degradation of performance across various domains as a result of aging. Indeed, previous findings have suggested that engagement in various lifestyle activities, including dance, are associated with a lower risk of cognitive decline in older adults (Verghese et al., 2003). However, conflicting results were reported by Verghese (2006), who compared motor and cognitive performances between older social dancers and non-dancers, and found that cognitive test performances did not differ between the two groups. Yet, observational studies do not shed light on the potential use of dance for improving cognition. Kattenstroth et al. (2013) recently addressed this by investigating the effects of a dancing intervention on older healthy adults. The experimental group consisted of older adults participating in a 6 months dance class, 1 h per week, who were compared to a matched control group. Results showed that participation in a dance class, even for only 1 h per week for 6 months, was able to induce positive effects across various domains. This included significant improvements being found in posture, reaction times, tactile and motor performances, attention, and across various cognitive domains. Improvements were also greatest in adults who had the poorest performances across the various domains tested before the dance classes took place, suggesting that dance was most effective in those with the greatest impairments prior to the intervention. Unfortunately, there was no measurement of the long lasting improvements of the dance class after the intervention had ceased. Other studies have also investigated the effects of dance training on cognition in older adults, but have only focused on specific cognitive domains. For instance, Coubard et al. (2011), with an interest on attention in older adults, compared approximately 6 months worth of training in dance classes to Tai Chi and fall prevention programs, and reported that only the dance group showed any improvement on attentional control. Kimura and Hozumi (2012) compared the effects of freestyle and combined dancing on task switching reaction time performance after a single session in healthy older adults, and reported that the combined dancing, by virtue of a decrease in switch cost post-dance class, influenced executive functioning, unlike the freestyle dance. However, no control group was included, and the findings, being based on a single session, cannot be used to interpret the impact of long term dance interventions on general cognition. Thus, although preliminary findings suggest that dancing can influence cognition, there remains insufficient evidence to thoroughly support such a notion.

Dancing interventions have also been used with various neurological populations, aiding with both physical and cognitive impairments. Arguably the most popular application of dance therapy has been with Parkinson’s patients, with specific interest on its impact on physical functionality. In regards to aiding with physical impairments, dancing has been noted to include many of the key features recommended in physical therapy for Parkinson’s (Keus et al., 2007; Earhart, 2009), including the use of external cues (including through music and movement of partners), teaching of specific movement strategies, incorporation of balance exercises, as well as acting as an aerobic exercise (Earhart, 2009). Indeed, dancing in those with Parkinson’s has been found to lead to significant improvements across various physical domains that are commonly impaired, including balance, gait, rigidity, upper extremity functioning and functional mobility (Earhart, 2009; Hackney and Earhart, 2009, 2010; Heiberger et al., 2011; Duncan and Earhart, 2012). Although Parkinson’s is primarily described as a movement disorder, it is also associated with impairments in cognition, particularly in executive functioning (Petzinger et al., 2013). However, dance studies involving Parkinson’s patients have primarily focused on the potential benefits on physical impairments, and less so on cognition. In a recent study by McKee and Hackney (2013), Parkinson’s patients were either enrolled into Tango dance lessons or an educational program, which was designed to include extensive socialization and interactions. In regards to cognition, those who participated in dance lessons were found to have improved in spatial cognition as well as executive functioning in comparison to the educational program controls. Gains were also maintained ∼10–12 weeks post-intervention. Improvements were suggested to be related to the motor training aspect of dance, since although social interactions may influence the efficacy of rehabilitation programs, such interactions were believed to be similar between the dance and educational group. Although improvements in executive functioning may in part be explained by dance acting as an aerobic exercise (Colcombe and Kramer, 2003), improvements in spatial cognition were speculated to be related to the dance lessons consisting of structured motor components that required memory of steps and directions, as well as awareness of spatial relationships and patterns in the environment (McKee and Hackney, 2013). Thus, regardless of substantial support for the use of dance in aiding with physical and functional impairments in Parkinson’s patients, evidence to support its use for improving cognition remains limited.

In regards to the use of dance to improve both physical and cognitive functioning in stroke and dementia patients, research remains sparse. Berrol et al. (1997) examined the effects of a 5 weeks, twice a week, dance class intervention for older stroke and traumatic brain injury patients. The dance intervention was tailored with psychosocial elements in mind, focusing on dance as not only an exercise, but as a creative and social activity. Significant improvements in the dance group were found for balance, self report scores related to social interactions, as well as in cognition, specifically on decision making and short term memory domains. A stroke related case study by Hackney et al. (2012) reported improvements on various physical domains, including balance, mobility and endurance as a result of dancing, although effects on cognition were not investigated. In regards to dementia related populations, Hokkanen et al. (2003) reported in a pilot study that cognition in Alzheimer’s patients did not change following a 16 week long dance intervention. Nonetheless, these results do not necessarily mean that dance did not have a positive effect. Rather, this may have implied that participation in dance class prevented the further decline of cognition associated with Alzheimer’s. A more recent study by Hokkanen et al. (2008) compared dance therapy in Alzheimer’s patients, with the inclusion of both a dance and control group. The dance class was held once a week, for 9 weeks in total. The dance group was found to have improved in specific cognitive domains, including that of visuospatial ability and planning, in comparison to the control group, in which cognitive performances, depending on the domain, remained either stable or declined. Van de Winckel et al. (2004) also reported that dance classes led to significant improvements in cognition in those with dementia. It is important to note that in this study, participation in the dance class was extensive, with sessions being held on a daily basis for 3 months. These findings are in agreement with those of Hokkanen et al. (2008), in the context that dance interventions for older adults with Alzheimer’s or dementia may be capable of improving cognition, rather than simply preventing or delaying decline. Similar to findings in healthy and other clinical groups, dancing has also been shown to improve physical functionality, such as strength, balance and gait, in those with Alzheimer’s (Abreu and Hartley, 2013).

Undoubtedly, research on dance as a tool for neurorehabilitation is still in its infancy. Yet, with the current evidence limited, particularly for its potential use in aiding with cognition in both healthy and neurological groups, there are still preliminary findings suggesting that dance can have a positive influence on both cognitive and physical functioning.

## CONCLUDING REMARKS

The number of people who will be affected by neurological disorders is expected to increase in the upcoming decades (World Health Organization, 2006). With issues in efficacy of current surgical and pharmacological treatments, as well as conventional rehabilitative therapies, new therapies are needed. As discussed, physical and musical therapies have been applied for aiding with both motor and cognitive impairments across various neurological groups. However, they also have inherent limits as discussed, creating the need to explore for other alternatives. With its multimodal nature due to simultaneously combining physical and cognitive activity, dance may offer a unique activity that can not only help with physical impairments, but cognitive as well. However, there are some important limitations in regards to the use of dance. Similar to other physical exercises, the recommendation of dance may be limited to those who are physically able; this may exclude patients who are in the most need of aid with physical and cognitive functioning. There may also be an issue with how dance is perceived as a potential treatment. Activities, such as dance, even if prescribed by health professionals, may be seen as recreational and less as a neurorehabilitative program. This perception may lead to some not recognizing the clinical value, and ultimately deciding not to participate (Chao et al., 2000).

With the lack of literature on the use of dance for neurorehabilitation, the future undertaking of certain research directions may be imperative to establish its efficacy. Research thus far has shown support for the use of dance in improving various physical domains. However, there is a lack of research on how dance can improve cognition, something that must be addressed if dance is to be promoted as a tool for neurorehabilitation. Future work with dance interventions, in both healthy and neurological groups, may strengthen the literature through the conducting of randomized controlled trials to investigate the impact of dance on cognition. Such interventions could consist of comparing dance classes to other activities, such as physical exercise or musical therapies, to address whether dance holds any unique therapeutic value for physical and cognitive functioning. As suggested earlier, the combination of physical activity with music may lead to an interaction effect, allowing dance to improve cognition in a unique manner compared to physical or musical activities alone; however, studies are needed to substantiate such claims. Investigating the potential impact of dance interventions may also be aided through the use of neuroimaging techniques, which as discussed, have been used to help elucidate how physical exercise and musical activities can enhance neuroplasticity and cognition; as of now, no known studies have been published which investigate changes to the brain as a result of dance interventions. There is also the issue of just what type of dance should be used; usually, the dance classes’ style of dance has been dependent on the instructor and their feeling of what the capabilities are of the participants. Dancing comes in various forms and styles, and even though they share similar traits, they also differ in many ways. However, at this point, focus should just be placed on the efficacy of dancing in general. A future line of research may focus on the efficacy of different types of dances in order to determine which may provide the greatest benefits. Additionally, research may be done in hopes of disentangling the multiple components of dance, such as music, verbal instructions, guided movements learned through visual demonstration, partnered dance either by leading or following, movement through space, and social interactions, in order to get a better sense of which of these may be primarily responsible for the positive changes associated with dance.

Dance, with its multimodal nature, may offer a unique method to address both physical and cognitive impairments in various neurological groups. In comparison to physical and musical therapies, we do not propose that dance undoubtedly holds the greatest impact of the three. Rather, it is most likely that each therapy holds its own clinical value unique to itself, as well as limitations. Thus, depending on the circumstances, dance may be a more appropriate and beneficial alternative therapy compared to others. Dance may also be able to overcome more practical issues that other therapies may face in their application. This can include it being a low cost therapy that can be done almost anywhere, thanks to little to no equipment needed. It may also, to some extent, avoid adherence issues due to it being a social and enjoyable activity. Thus, dance should be further investigated for its potential use as a tool to not only help with aging gracefully, but more importantly, to help those with their fight against neurological disorders.

## Conflict of Interest Statement

The authors declare that the research was conducted in the absence of any commercial or financial relationships that could be construed as a potential conflict of interest.
